# Habit Formation and the Effect of Repeated Stress Exposures on Cognitive Flexibility Learning in Horses

**DOI:** 10.3390/ani12202818

**Published:** 2022-10-18

**Authors:** Cathrynne Henshall, Hayley Randle, Nidhish Francis, Rafael Freire

**Affiliations:** School of Environmental, Agricultural and Veterinary Sciences, Charles Sturt University, Wagga Wagga, NSW 2650, Australia

**Keywords:** cognitive flexibility, habit, repeated stress, equine cognition, reversal learning, BDNF, cortisol, equine ethology

## Abstract

**Simple Summary:**

Horse training exposes horses to many difficult learning challenges, including tasks that run counter to their natural behaviour and research from neuroscience can be used to understand how horses respond to these learning challenges. Horses can also quickly develop habits, however if the habit is undesirable, the horse may be subjected to retraining where it must learn to suppress the unwanted behaviour and learn a new response. This is called cognitive flexibility and is underpinned by complex neural processes and can be impaired by exposure to repeated or chronic stress. We trained horses to acquire habit-like responses in one of two industry-style aversive instrumental learning scenarios (moving away from the stimulus-instinctual-IS or moving towards the stimulus-non-instinctual-NIS) and evaluated the effect of repeated stress exposures on their cognitive flexibility in a reversal task. We measured heart rate, salivary cortisol and serum brain derived neurotrophic factor to infer possible neural correlates of the learning outcomes. Horses trained in the NIS task took longer to learn task than the horses trained in the IS task, however they were quicker to learn the IS task during the reversal. There was no significant effect of the stress exposures on cognitive flexibility, however the stress did make horses more sensitive to the aversive training stimuli. These results provide guidance for trainers to adapt their practices when training tasks that are difficult for horses to learn and during retraining. In particular, even when horses have been trained to approach aversive stimuli they can quickly learn to avoid them. This has implications for retraining unwanted responses during aversive situations such as trailer loading.

**Abstract:**

Horse training exposes horses to an array of cognitive and ethological challenges. Horses are routinely required to perform behaviours that are not aligned to aspects of their ethology, which may delay learning. While horses readily form habits during training, not all of these responses are considered desirable, resulting in the horse being subject to retraining. This is a form of cognitive flexibility and is critical to the extinction of habits and the learning of new responses. It is underpinned by complex neural processes which can be impaired by chronic or repeated stress. Domestic horses may be repeatedly exposed to multiples stressors. The potential contribution of stress impairments of cognitive flexibility to apparent training failures is not well understood, however research from neuroscience can be used to understand horses’ responses to training. We trained horses to acquire habit-like responses in one of two industry-style aversive instrumental learning scenarios (moving away from the stimulus-instinctual or moving towards the stimulus-non-instinctual) and evaluated the effect of repeated stress exposures on their cognitive flexibility in a reversal task. We measured heart rate as a proxy for noradrenaline release, salivary cortisol and serum Brain Derived Neurotrophic Factor (BDNF) to infer possible neural correlates of the learning outcomes. The instinctual task which aligned with innate equine escape responses to aversive stimuli was acquired significantly faster than the non-instinctual task during both learning phases, however contrary to expectations, the repeated stress exposure did not impair the reversal learning. We report a preliminary finding that serum BDNF and salivary cortisol concentrations in horses are positively correlated. The ethological salience of training tasks and cognitive flexibility learning can significantly affect learning in horses and trainers should adapt their practices where such tasks challenge innate equine behaviour.

## 1. Introduction

The training of horses for handling, riding, competition and transport exposes them to an array of cognitive and ethological challenges [1]. In addition, horses are frequently exposed to various forms of stress including multiple, repeated and chronic stressors that in other species have been shown to impair learning. Common learning challenges horses experience include tasks that are antithetical to their ethology, such as approaching rather than avoiding aversive stimuli such as transport vehicles or tasks that require they forget previous habits and learn new ones, such as occurs when they are sent for ‘retraining’ of undesirable habits. Training failures, where horses either fail to acquire the ethologically challenging learning task, rapidly acquire unwanted habits, or experience difficulty undergoing extinction of an unwanted habit during retraining, are commonly reported in industry and a there is wide variation in the advice given to owners about how to resolve these issues [2]. Evidence from other species suggests that cognitive and ethological challenges during learning are influenced by complex neural processes that are vulnerable to impairment by chronic stress [3]. The identification of factors such as stress that may affect horses’ learning during cognitive and ethological learning challenges, and in particular the inference of potential neural influences on learning behaviour could assist horse owners and trainers to improve their practice, benefiting horse welfare and human safety.

The ethology of horses has been widely studied and their responses to aversive stimuli have been well characterised [4,5]. Horses are characterised as flight animals whose genetically hard wired [6] species specific defence reaction (SSDR) [7] is to escape from or avoid stimuli or places that they have evolved to find threatening to their safety, such as poorly lit enclosed spaces and stimuli that are novel, unpredictable or painful [4]. SSDRs are believed to be underpinned by what have been termed ‘instinctive’ neural and associated emotional networks that facilitate behavioural responses to threats without the necessity of prior learning [8]. However, SSDRs can be modified via learning in response to environmental conditions, as the rich literature in experimental avoidance learning and fear conditioning demonstrates. In the domestic horse, SSDR are routinely modified by training such that horses learn to approach aversive stimuli rather than escape or avoid them [9]. With appropriate techniques, horses can be successfully trained to approach and habituate to these stimuli, such as occurs during routine transport [10] or during policing work [11]. However, not all horse training is successful, and difficulties loading horses onto transport vehicles are commonly reported [12]. In addition, even where horses have been successfully trained or habituated to approach aversive stimuli, they can rapidly acquire avoidance or escape responses, including dangerous behaviours such as rearing and bolting [13]. These SSDRs are likely to occur under the influence of a defensive neural network that facilitates adrenaline mediated flight responses [14]. SSDRs in horses can be rapidly acquired and can be resistant to extinction likely due to noradrenaline and cortisol mediated consolidation in the basolateral and central nuclei of the amygdala which regulate fear memories [15,16]. Consequently, current training advice is to use methods that minimise fear responses during the training of responses that have the potential to elicit instinctive escape responses [17]. There are few experimental data comparing rates of acquisition of trained responses based on their alignment with the horse’s innate escape behaviour which could inform owners about how to enhance learning of these types of tasks.

Another cognitive challenge that horses are exposed to occurs when they undergo retraining for unwanted behaviour. Owners commonly seek professional assistance with ‘retraining’ the horse out of undesirable behavior [18]. During retraining the horse must learn to suppress the unwanted habits and to develop new ones. This process is known as cognitive flexibility and requires individuals to modify their behaviour in response to changes in the environment or in the case of horses, in response to the actions of the trainer, who may punish a previously reinforced behaviour [19]. Cognitive flexibility is underpinned by competition and cooperation between two neural networks believed to facilitate instrumental learning and the development of habits; the flexible, goal-directed network and the less flexible, stimulus-response (SR), habit network [20]. The goal-directed network is responsible for acquisition in instrumental learning, in which actions and their consequences determine whether behaviour is repeated or supressed [21,22,23]. With repetition, the SR neural network assumes control of responding [24,25] in part by inhibiting activity in the goal-directed network [26,27,28,29]. When the SR network is dominant, responses become habitual and may become resistant to extinction so that even when the response no longer delivers the expected outcome, it will continue to be elicited by the stimulus [30,31]. It should be noted that the evidence for this shift has been obtained from appetitive instrumental learning tasks and although there are suggestions that aversive instrumental learning is also underpinned by similar patterns of activity in these networks (reviewed in Manning et al. [32] Cain, [33] LeDoux and Daw, [34] there is little experimental evidence in this area. In a rare example, O’Malley and Brunning [35] reported that rats whose behaviour was highly habitual made more perseverative errors in an aversive spatial reversal learning task.

In the common scenario in which horses are sent to a trainer to undergo retraining, the desired goal of the training is that unwanted habitual responses to cues are extinguished and new (desired) habitual responses are learned, so that the horse ‘automatically’ offers the (new) desired response and the unwanted response is no longer performed. To facilitate this, the trainer alters the contingency, so that previously reinforced behaviour is punished, which in turn motivates the horse to trial new responses to the cue to escape the punishment of the old response. In order for this to occur there must be a shift in the activity of the neural networks controlling behavioural responses to training stimuli, so that its flexible goal-directed network is able to reassert primacy over learning to enable novel behaviours to be trialled and learnt [19]. This form of cognitive flexibility requires the generation of two memories: the inhibition and eventual extinction of the original response (extinction of the existing SR association), and the new goal-directed response memory [36], so that the new response can be performed and with repetition, become the new habit.

In early retraining, the demand for cognitive flexibility is greatest as a result of the simultaneous inhibition of the unwanted habit and the learning of the new response [24]. The horse is likely to experience conflict about which response to make, because its previously learned prediction about how to achieve a beneficial outcome (escape or avoidance of aversive training stimuli) is no longer accurate [32]. This conflict, conceptualised as a prediction error, is believed to motivate learning in situations where the behavioural solution to a problem is not clear [37] and is signalled by changing patterns of neurotransmitter release, particularly dopamine [38]. Dopamine release associated with learning outcomes is critical for signalling the valence (better or worse than expected) and value (high or low) of prediction errors to generate the motivational drive to adapt to the new situation [39,40,41]. Of particular relevance here is that worse than expected outcomes are signalled by a reduction in dopamine release [42]. A failure to update a prediction error when the situation has changed can mean the horse persists in making the original response despite receiving punishment for doing so, which may have negative effects on their welfare.

Cognitive flexibility in horses has been examined using appetitive spatial and visual discrimination instrumental learning tasks in horses affected by stereotypies [43,44,45] as well as non-affected horses [46,47]. Among the latter, those assessed as having a fearful temperament [47], or having been previously exposed to a stressor in the learning context [46], were shown to have impaired cognitive flexibility. This is critically important because aversive instrumental learning predominates in horse training [48] and consequently there is a gap in knowledge in regards to aversively motivated cognitive flexibility in domestic horses.

Evidence from non-equine species demonstrates that exposure to repeated, multiple or chronic stressors can impair cognitive flexibility in appetitive instrumental learning, and lead to disruption of the individual’s ability to adapt to change [3,49,50]. Chronic stress may adversely affect the function and morphology of the relevant brain regions responsible for cognitive flexibility learning [51,52]. These alterations occur as a result of chronic stress effects on the release and activity of neurotransmitters such as glucocorticoids, noradrenaline, and brain derived neurotrophic factor (BDNF), favouring habitual responding that can impair cognitive flexibility [3,50,53]. In particular, rodents exposed to unpredictable chronic stress has been shown to impair cognitive flexibility in some but not all appetitive learning protocols [3] and chronic stress is also associated with blunted sensitivity in dopamine driven prediction error signalling in relation to identifying beneficial outcomes [54]. In industry, horses may be exposed to situations that elicit stress responses over extended periods [55,56,57,58], however there are relatively little data on the effects of repeated or extended exposures to stressors on equine behaviour, learning or cognitive flexibility [55,56,57].

Currently, there are no methods for exploring relationships between neurotransmitter concentrations in specific brain regions and cognitive or behavioural outputs in behaving horses and limited extra-cranial proxies such as EEG or spontaneous eye blink rate [59,60]. However, the inference of neurobiological processes that may underpin cognitive outputs from peripheral concentrations of neurotransmitters (for example [61]) or from findings in other species such as rodents [62] provides a mechanism to explore these issues. Proxies for neurotransmitter activity that have been measured in horses include salivary cortisol and heart rate (HR). Salivary cortisol is a validated measure of systemic hypothalamic pituitary adrenal (HPA) axis activity in horses [63] and HR can provide a proxy for sympathetic nervous system activity where peripheral adrenaline and noradrenaline concentrations increase in alignment with HR [64,65,66,67,68]. Recently, peripheral concentrations of the neurotrophin BDNF have been measured in equine serum [69]. BDNF is an abundantly expressed neurotrophin which along with activity in its receptors, facilitates learning acquisition and memory consolidation through a range of synaptic plasticity and neurogenic processes [70,71,72,73]. There is growing evidence that under stress conditions, glucocorticoid-BDNF interactions [74,75,76] lead to the persistence of SR behaviour at the expense of goal-directed learning, impairing cognitive flexibility [77]. In swine and rodents, serum and hippocampal BDNF concentrations have been shown to correlate [78,79]. In addition, peripheral BDNF concentrations have been correlated to learning outputs in some but not all in human cognition studies [80].

Anecdotally horse owners and trainers report difficulties training horses in tasks that engage instinctive defensive or escape responses, as well as retraining unwanted habits [48]. Trainers may perceive the horse’s failure to rapidly acquire the desired response as a deliberate disobedience, resulting in punishment or an increase in the intensity or frequency with which aversive training stimuli are applied, a situation that may have negative consequences for the horse’s welfare [81]. As horses are routinely exposed to stressors of varying intensities and durations, the elucidation of factors that impair cognitive flexibility in horses, including putative neurophysiological factors, would benefit both owners and horses.

We compared the rate of habit acquisition of two learning tasks that differed in their alignment with equine ethology, specifically escape responses, and then examined the effects of six days of repeated, multiple stress exposures on cognitive flexibility learning acquisition. HR, salivary cortisol and serum BDNF concentrations were analysed to assess if these proxies were associated with cognitive performance during learning. We hypothesised the alignment of the task with the horses’ innate escape behaviour would affect the rate of habit acquisition. We also hypothesised that the stress treatment would impair the acquisition of the cognitive flexibility as a result of stress induced alterations to brain networks and regions responsible for cognitive flexibility.

## 2. Materials and Methods

### 2.1. Animals and Management

A convenience sample of twelve horses of mixed breeds (Table 1), age range 7–17 years, (mean 12.08 years) and sex (eight mares and four geldings) were recruited from private owners. During the recruitment process, horses were given two taps on the gluteal area of the hindquarters level with the hip joint with 1.1 m long dressage whip to test for prior training in the target response. Horses that did not make any locomotory reaction to the whip were included in the study and those that did respond were excluded.

All but one of the recruited horses had been trained for riding but none had been ridden in the six months prior to the study. Externally owned horses were brought to the experimental location 14 days prior to the commencement of the study. The horses were kept at in two mixed sex groups and maintained on pasture and supplementary lucerne and cereal hay in large paddocks (20 ha) overnight and smaller paddocks (2 ha) adjacent the testing area on test days.

### 2.2. Infrastructure/Stimuli

All data collection occurred in the open air. The horses were prepared for each daily activity in an area adjacent to the holding paddocks which was also the location for the Pre-Test phase (PT) and the Control (C) treatments. The learning and memory testing area was an adjacent 12 m × 6 m rectangle bordered by coloured jump poles on the ground.

There were three stressor types: social isolation (SI), novel object (NO) and multimodal stressor (MS) (details supplementary Table S1). The SI was a 10 m × 4 m yard, located 300 m from the PT area and visually isolated from other horses. The NO and MS exposures occurred in a 20 m rubber lined roundyard located 50 m from the PT area. The NO was a trio of exercise balls ranging in diameter from 70 cm to 1.5 m diameter which were manipulated to be unpredictable and uncontrollable by an experimenter inside the roundyard. The MS comprised a combination of aversive sounds (dogs barking, gunshot, sirens, applause and human shouting which were amplified by a portable amplifier on the maximum volume setting and a 30 cm × 20 cm × 15 cm electric radio-controlled car (RCC). The sounds and RCC were manipulated by two experimenters standing outside the roundyard.

### 2.3. Data Collection

All sessions with the exception of PT were recorded with a Sony CX650 video camera (Sony Corporation, Tokyo, Japan). Learning related data were manually recorded during each trial and later transcribed into MS Excel. Cardiac data were collected via a Polar V800 heart rate monitor (HRM) set to the R-R mode, with a Polar H10 sensor attached to Polar equine electrodes (Polar Electro Oy, Finland). Two identical kits were used to allow simultaneous recording of pairs of horses during the PT session and control treatment and the data were uploaded daily to Polar Flow via the Polar Sync phone app for Android. 

### 2.4. Physiological Samples

Saliva samples were collected with a Salivette saliva collection device (Sarstdet, Nümbrecht, Germany) [82]. Samples were held on ice immediately after collection and then centrifuged at 1000× *g* for 10 min and aliquoted into 1.5 mL Eppendorf^TM^ tubes, and then frozen at −20 °C until analysis.

Serum samples were collected via venepuncture into BD SSII Vacutainers^TM^. During the PT phase two horses showed extreme reactions to venepuncture (one from each treatment group) and were excluded from further blood sampling, leaving 10 horses from which samples were collected. Samples were clotted for approximately one hour before centrifuging at 1300× *g* after which 1 mL of each sample was aliquoted for storing on dry ice. The ambient temperature during clotting ranged from 18–30 °C. At the conclusion of the experiment all samples were transferred to a −80 °C freezer until analysis.

### 2.5. Experimental Sequence

#### 2.5.1. Daily Preparation

Horses were prepared in pairs to reduce separation anxiety prior to testing. Each horse was given a light grooming and the HRM was fitted with the electrodes held in place via an elasticised surcingle.

- Day 1 Pre-test (PT)

Between 7.30 am and 11 am, pairs of horses were tethered in visual and tactile contact and left undisturbed for 15 min. At the conclusion of the 15 min saliva and serum samples were collected.

- Days 2–3 Learning
- Training procedure-original learning (OL)

**Figure 1:**
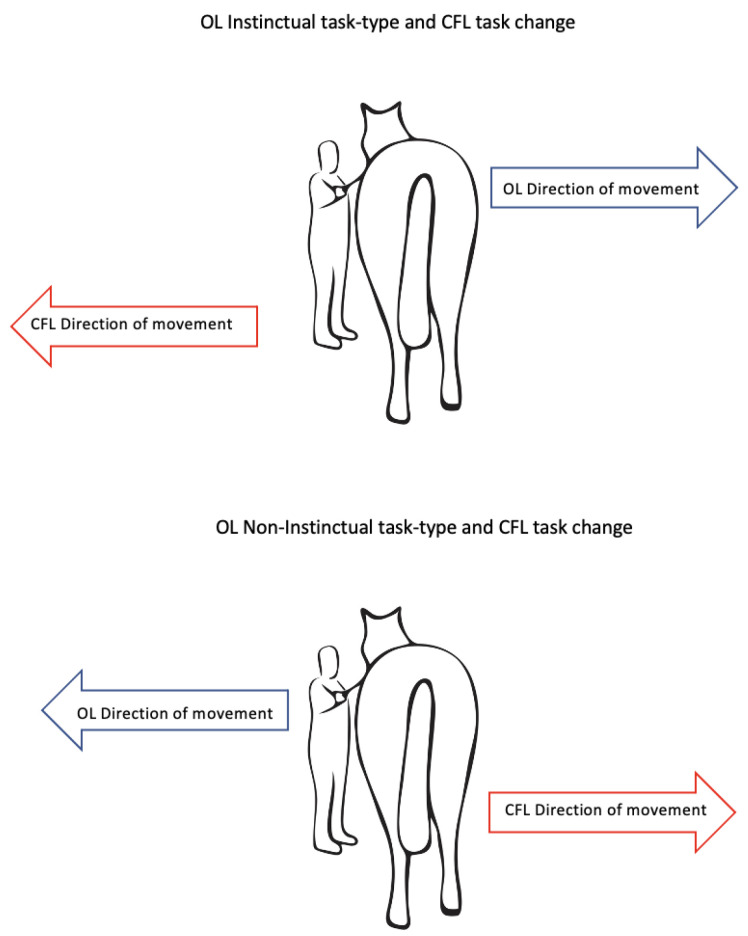
Diagram of the direction of movement for the IS and NIS task types during original learning (OL) and then the direction change during the cognitive flexibility learning (CFL).

The horses were randomly assigned to instinctual (IS) or non-instinctual (NIS) task-type groups, based on order in which they were caught prior to the PT session, with the first horse of the experiment allocated to the IS task type and thereafter each horse that followed alternated between the NI and back to the IS tasks until all horses had undergone the first OL session. This order was maintained for all subsequent sessions of the experiment The training commenced as follows. The trainer (author CH) stood on the right side of the horse, level with its shoulder. A 1.10 m long dressage whip was held in their left hand, and raised to the tapping position (level with the horse’s hip joint) and held in position next to but not touching the horse for 2 s. If the horse did not respond, gentle tapping in steady rhythm commenced until the horse made an attempt to move the hindquarters away from the whip (IS task/group) or towards the whip (NIS task/group), (Figure 1). The same intensity and frequency of taps was maintained until the horse made a locomotory response. The next trial commenced immediately the horse came to a standstill after making the locomotory response. In the first session the tapping ceased immediately the horse lifted a hindleg towards the midline, even if the movement was small. In the second and subsequent trials the tapping continued after the first step to encourage the horse to perform a second step which was the correct response. The tapping ceased and the whip tip lowered to ground level, when in the opinion of the trainer the second step was in the process of being performed. In some instances, the horse did not make a full second step by the time the tapping had ceased or the horse took more than two steps. Responses were initially scored from 1 to 3, with a score of 1 denoting half or one full sideways step, 2 denoting two sideways steps and 3 denoting three or more sideways steps.

There were four 25 trials original learning sessions (OL1–4). Horses were tested in the same order for each session as per Table 1. Sessions were held twice daily (AM and PM) for two days with a four hour gap between sessions. Serum samples were collected at the conclusion of the OL1 and OL4 and saliva samples were collected 10 min later as per the timing of the physiology of cortisol release [83].

At the conclusion of the OL, the number of taps each horse received was compared as a measure of the learning efficiency of the horse to ensure the treatment groups contained a representative sample of efficient and inefficient learners. A Kruskal–Wallis independent samples test with horse as the grouping variable was used as the data were not parametric (Shapiro–Wilk: *p* < 0.05). There were no significant differences between the horses and they were randomly allocated into the stress (S) or control (C) treatment with 6 x IS and NIS horses in each treatment.

- Days 3–8: Treatment

**Figure 2:**
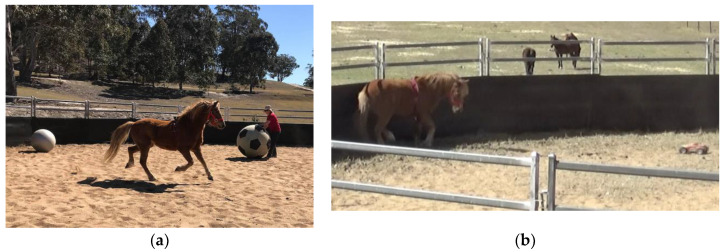
(**a**). Novel object stress treatment. (**b**). Multi-modal stress treatment-radio controlled car.

There were six daily treatment events (T1–6) of 15 min in duration (details supplementary Table S1). A single stress type was applied each day: social isolation followed by the novel objects (Figure 2a) and then multi-modal stressors (Figure 2b). This sequence was repeated so that each horse was exposed to each stressor twice. The C horses were tethered in visual contact with a familiar companion and left undisturbed. At the conclusion of treatment sessions 1, 3 & 6 saliva and serum samples were taken.

- Day 9: Cognitive Flexibility Learning (CFL)

On the day after the last treatment, the horses underwent CFL training in the learning area. The training method was identical to the first OL session however the type of task the horse had to perform was reversed (Figure 2), that is, IS horses had to learn the NIS task (moving toward the whip) and the NIS horses had to learn the IS task (moving away from the whip). Pressure on the leadrope was used where required to prevent the horse from trialling forward movement instead of the sideways movement of the new task. The tapping was ceased when the horse made a single step in the correct direction for their CFL task. At the conclusion of the first 15 trials the horse was given a 120 s break and then a second set of 15 trials. Serum samples were taken at the conclusion of the session and due to an error, the saliva samples were also collected immediately after the end of the session rather than 10 min afterwards as per the OL collection schedule.

#### 2.5.2. Biochemical Analysis

- Cortisol

Undiluted saliva samples were analysed with a Salimetrics Salivary Cortisol ELISA kit (State College, PA, USA) as per manufacturer instructions. The plates were read in a BioTek BTK-800TSI plate reader (BioTek, Winooski, VT) at 450 nm with a 490 nm correction filter. Samples were assayed in duplicate with an intra assay COV range of 2–5% and an inter assay COV of 3.3 ± 1.25%. Samples with a COV of >10% were re-assayed using the same procedure. Concentrations are reported as ng/mL.

- Brain Derived Neurotrophic Factor

Samples were assayed with a Total BDNF Quantikine ELISA (R&D Systems Inc, Catalogue item DBNT00, Minneapolis, MN, USA) as per the kit instructions. Pilot testing was undertaken to determine the optimum sample dilution, at 0, 2, 4 and 8 × dilutions and to compare equine BDNF levels with rat and human sera. The mean concentrations of equine BDNF (14.647 pg/mL) were found to be substantially lower than concentrations in the pooled human (33,839 pg/mL) and rat (5301 pg/mL) sera. At dilutions greater than 4 × BDNF concentrations were lower than the minimum detectable concentration of the kit (0.997 pg/mL). Samples diluted at 0, 2 and 4 × were within 0 and the lowest standard (15.6 pg/mL). Based on this assessment the samples were assayed undiluted. Samples were thawed at 4 °C and assayed immediately. The plates were read with a BioTek BTK-800TSI plate reader (BioTek, Winooski, VT, USA) at 540 nm with a 570 nm correction filter. Samples were assayed in duplicate with an average intra-assay COV of 6% and inter assay COV of 6%. Concentrations are reported in pg/mL.

- HR data Analysis.

The raw HR data were extracted from Polar Flow as text files and imported to Kubios HRV Premium, ver. 3.2.0, (Kubios Oy, Kuopio, Eastern Finland) to covert the raw R-R data to beats per minute (bpm) and correct artefacts. The automatic correction filter was applied [82] and the resultant data then exported to an MS Excel file.

- Statistical analysis

The data were analysed using SPSS (IBM, Amarok, NY release 27, and SAS Online (Cary, NC, USA). For all analyses, the OL task-type (IS or NIS) was taken as the reference task-type. The learning efficiency of individual horses as determined by the number of taps applied during the OL sessions was assessed to facilitate allocation of horses to the treatment groups, to ensure that each treatment group contained a balance of efficient and inefficient learners. The data was normally distributed (Shapiro–Wilk, *p* < 0.05) and the total number of whip taps applied to individual horses during the four OL sessions was calculated and analysed as a single count per horse with a Kruskal–Wallis Independent samples test (KW), with taps as the dependent variable and horse as the explanatory variable.

The learning response variables during the OL, CFL of correct response and size of response (whether greater than one step, >1 step, were not normally distributed and not able to be transformed to achieve normalisation. Consequently, they were converted to binary variables to facilitate analysis using a mixed model approach which enabled consideration of a range of factors and interactions that are not available in non-parametric tests. Predicted probabilities were calculated to facilitate interpretation of the statistical test results (descriptors of binary codes in supplementary Table S2). The analysis of the four OL sessions and the comparison of OL1 session to the CLF session were analysed with Generalised Linear Mixed Models with binary logistic regressions and the single CFL session was analysed with a with a full factorial General Linear Model. The last 5 trials of the CFL session were removed from the comparison with the OL1 session to ensure an equal number of trials were compared.

Pre-test physiology measurements were grouped into task-type and treatment groups and analysed with paired samples *t*-tests to assess if there were pre-existing differences prior to learning and treatment. HR and cortisol and BDNF concentrations during OL and treatment were assessed with a Linear Mixed Model (LMM) with either task-type and session or treatment group and session as fixed effects, horse as random effect and physiological value as the dependent variable. Effect of treatment and task-type on HR, BDNF and cortisol during the CFL were analysed using a full-factorial General Linear Model (GLM).

Spearman’s rank correlations were performed to assess if there were associations between the physiological indicators and learning performance in the OL and CFL and between physiological indicators during the treatment. Statistical significance was set at *p* < 0.05. Data are presented as Means ± SD or Means + [95% Confidence Intervals-CI].

## 3. Results

### 3.1. Pre-Test Physiology

There were no significant differences based on task-type group or treatment group for any physiological measure (HR, salivary cortisol, serum BDNF, data in supplementary Table S3).

### 3.2. Original Learning

**Figure 3:**
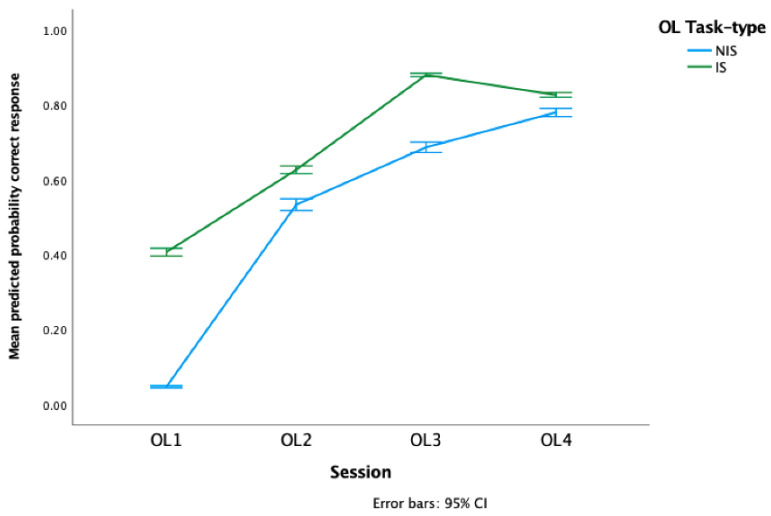
Mean predicted probability of a correct responses per trial during the original learning sessions. OL 1–4 = individual original learning sessions.

The probability of a correct response of two full steps significantly increased as the sessions progressed (*F*_3,1192_ = 61.90, *p* < 0.000001, Figure 3). The IS horses had a significantly higher probability of performing correct responses than the NIS horses overall (*F*_1,1192_ = 14.98, *p* = 0.0001), and there was a significant task-type*session interaction *F*_3,1192_ = 9.26 *p* < 0.00001). In addition, IS horses had a higher probability of making a larger locomotory responses (>1 step) than the NIS horses overall (Task-type: *F*_1,1190_ = 8.27, *p* = 0.004), particularly in the early sessions (task-type*session: *F*_3,1190_ = 87.58, *p* < 0.000001, full > 1 step variable statistics, data and supplementary Figure S1 in Supplementary Materials).

The duration of the first OL session was significantly longer than the other three OL sessions (*F*_3,40_ = 33.52, *p* < 0.000001, supplementary Figure S2). The task-type did not significantly influence the duration of learning (*F*_1,40_ = 0.69, *p* = 0.41) and there was no significant OL session*task-type interaction (*F*_3,40_ = 0.55, *p* = 0.11).

The number of taps applied decreased across the OL sessions (*F*_3,1192_ = 167.09, *p* < 0.000001, supplementary Figure S3). There was no significant difference between the task-type groups, (IS: 151.58 ± 212.48/all OL sessions, NIS: 227.67 ± 239.82/all OL sessions *F*_1,1192_ = 3.51, *p* = 0.06) and there was no session*task-type interaction (*F*_3,1192_ = 1.49, *p* = 0.22). The individual horses did not significantly differ in their learning efficiency based on the number of whip taps they received across the four OL sessions (KW:*X*^2^ _(11)_, 8.61, *p* = 0.66).

**Figure 4:**
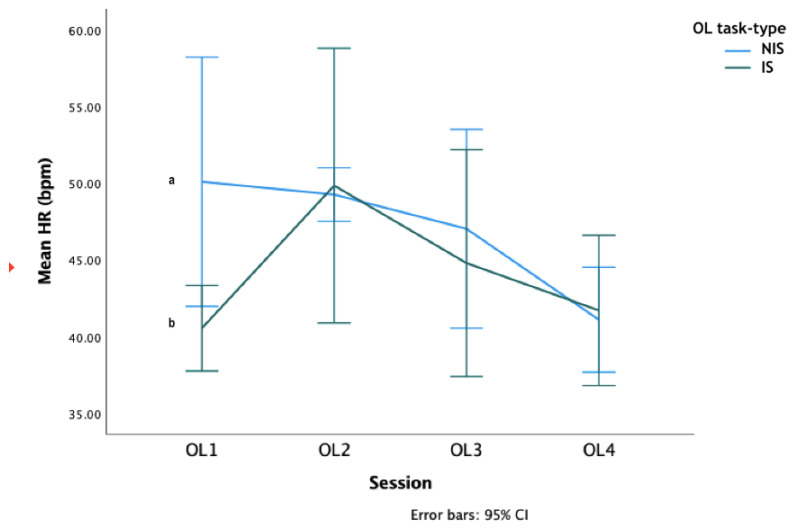
Mean HRs (bpm) during the original learning sessions. OL = original learning. Letters that differ, differ at *p* < 0.05.

HRs varied significantly across the OL sessions, with HRs increasing in OL1 and OL2 and decreasing in OL3 and OL4, (LMM: *F*_3,30_ = 6.49, *p* = 0.002, Figure 4). There were no differences between the HRs based on the task-type across the four learning sessions (LMM: *F*_1,10_ = 1.21, *p* = 0.29). The session*task-type interaction was however significant (LMM: *F*_3,30_ = 3.34, *p* = 0.032), with the NIS horses’ HRs significantly higher than the IS horses during the first learning session but not the other three sessions (GLM: OL1: *F*_1,10_ = 8.17 *p* = 0.017, OL2: *F*_1,10_ = 0.03 *p* = 0.87, OL3: *F*_1,10_ = 0.34 *p* = 0.57, OL4: *F*_1,10_ = 0.07 *p* = 0.8 Figure 4).

There were no significant differences in salivary cortisol concentrations between the task-type groups during the two sampled OL sessions (OL1 and OL4) (Means ng/mL: IS: 0.969 [0.909–1.1.029 95% CI],NIS:0.1.045 [0.975–1.115 95% CI] LMM:*F*_1,20_ = 3.78 *p* = 0.066), between sessions (LMM: *F*_1,20_ = 1.12 *p* = 0.30, or a significant session*task-type interaction (LMM: *F*_1,20_ = 1.19 *p* = 0.61).

There were also no significant differences in BDNF concentrations during the sampled OL sessions, (pg/mL: IS: 10.615 [4.125–17.106 95%CI], NIS: 13.727 [97.237–20.218 95% CI] LMM: *F*_1,8_ = 0.61 *p* = 0.46), nor was there a session (LMM: *F*_1,8_ = 2.02 *p* = 0.19) or significant task-type*session interaction (LMM: *F*_1,8_ = 0.72 *p* = 0.42).

### 3.3. Treatment

**Figure 5:**
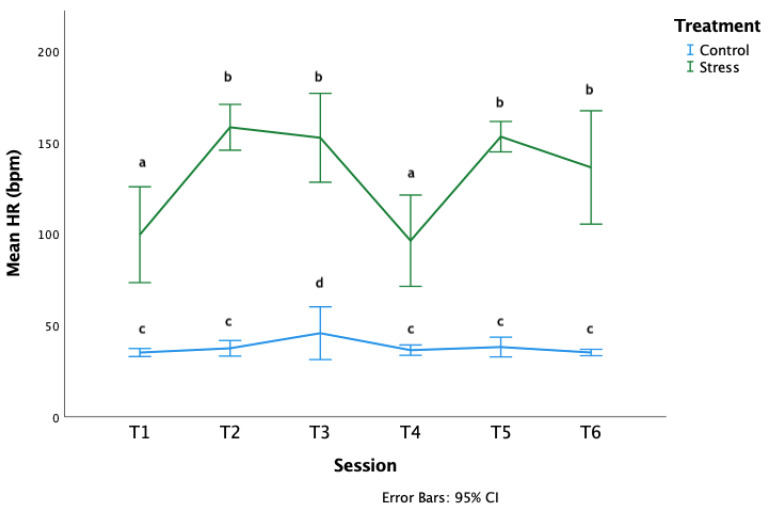
Mean heart rates (bpm) during the six treatment sessions. Letters which differ, differ significantly at *p* < 0.0001.

The HRs of the S horses during treatment were significantly higher than the HRs of C horses (LMM: *F*_1,60_ = 635.34 *p* < 0.000001 Figure 5). HRs also differed significantly between sessions (LMM: *F*_5,60_ = 10.75 *p* < 0.000001). The treatment*session interaction was also significant (LMM: *F*_5,60_ = 8.07, *p* = 0.000007): the S horses had lower HRs during social isolation stress (sessions 1 and 4) compared to the other sessions and the C group horses had higher HRs during the third treatment session compared to the other sessions.

**Figure 6:**
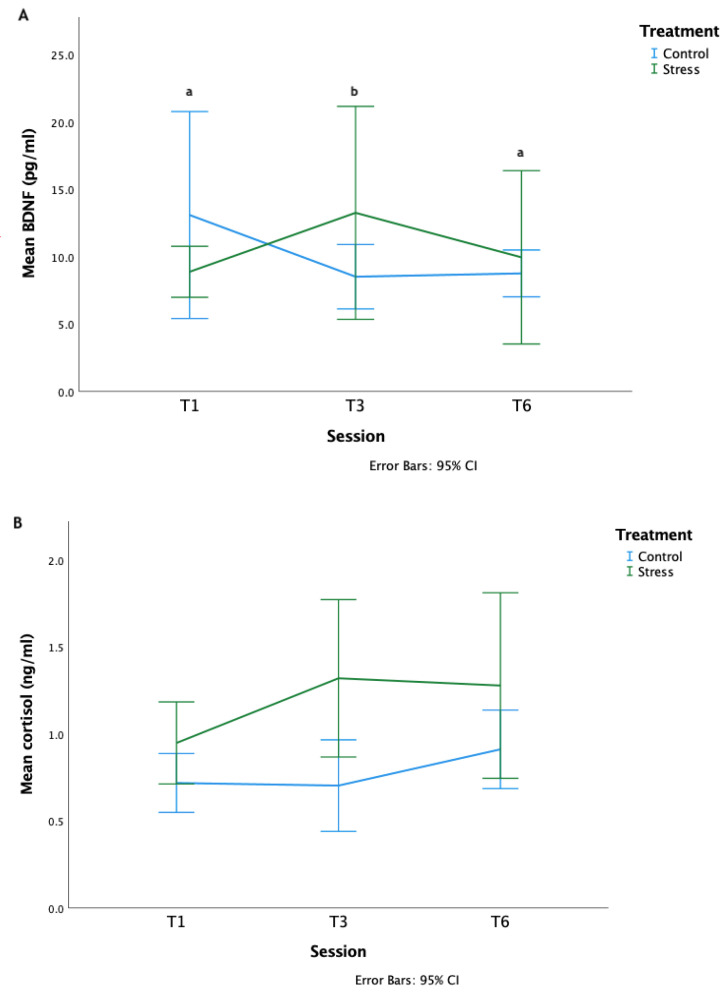
(**A**). Concentrations of BDNF (pg/mL) during the treatment. T = treatment session. Letters which differ, differ significantly at *p* < 0.01 between sessions. (**B**) Concentrations of salivary cortisol (ng/mL). T = treatment session.

There was no effect of the treatment on serum BDNF concentrations (LMM: *F*_1,8_ =0.67 *p*=0.435). However, concentrations did differ across the three sampled sessions with mean concentrations significantly lower in T3 than the other samples sessions (pg/mL—T1: 11.85 ± 5.92, T3: 8.82 ± 3.91, T6: 11.45 ± 5.18, LMM: *F*_2,16_ = 12.54 *p* = 0.001, Figure 6A). The session* treatment interaction was not significant (LMM: *F*_2,16_ = 0.75 *p* = 0.49).

The S horses’ salivary cortisol concentrations were significantly higher than the C horses, LMM: *F*_1,10_ = 9.28 *p* = 0.01 Figure 6B). There were no significant differences between phases (LMM: *F*_1,20_ = 2.75 *p* = 0.08) or session*treatment interaction (LMM: *F*_1,20_ = 1.48 *p* = 0.25).

### 3.4. Cognitive Flexibility Learning Acquisition

**Figure 7:**
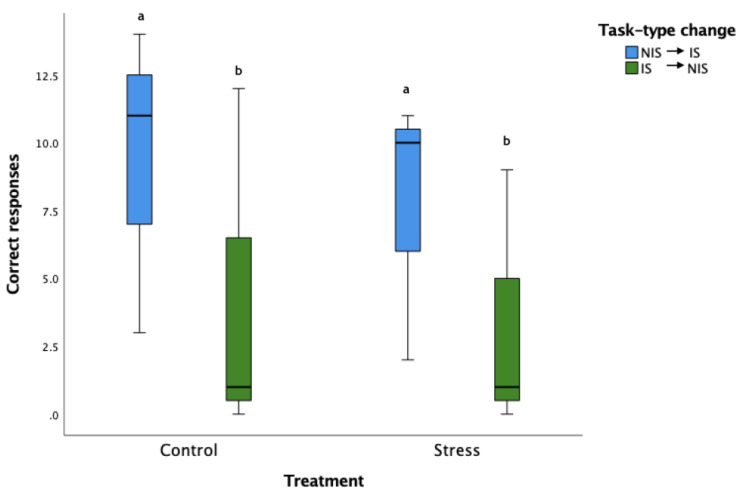
Box plot of correct responses during the cognitive flexibility learning session. Areas within boxes represent 25–75% quartiles, solid line within box represents median, whiskers represent minimum/maximum quartiles. In the cognitive flexibility phase, horses that were in the OL NIS group, were trained to perform the IS task and vice versa. Letters that differ, differ significantly at *p* < 0.01.

The horses that shifted from NIS to IS performed significantly more correct responses than horses shifting from IS to NIS (Means: NIS-IS: 8.5 ± 4.85, IS-NIS: 23:3.83 ± 5.27, *F*_1,356_ = 9.60 *p* = 0.002, Figure 7). There was no significant treatment effect on correct responses (Means: C: 6.83 ± 6.18, S: 5.50 ± 5.01, *F*_1,356_ = 0.27 *p* = 0.61) or a significant treatment*task-type interaction (*F*_1,356_ = 0.60 *p* = 0.44).

The S horses had significantly fewer taps applied per trial than the C horses (Means: C: 15.06 ± 16.92/trial, S: 13.81 ± 16.92/trial (*F*_1,356_ = 7.88, *p* = 0.005, as did the IS to NIS task horses compared to the NIS to IS task horses (IS to NIS task: 15.75 ± 16.90/trial, NIS to IS task: 12.63 ± 16.81/trial; *F*_1,356_ = 19.82, *p* = 0.00001, supplementary Figure S4). The treatment* task-type interaction was not significant (C IS to NIS task: 22.20 ± 16.26/trial, C NIS to IS task:11.34 ± 11.08/trial, S IS to NIS task: 14.17 ± 17.78/trial, S NIS to IS task: 9.74 ± 11.08/trial, *F*_1,356_ = 3.51, *p* = 0.06).

There was a significant effect of change in task-type and treatment*change in task-type interaction, but not treatment alone on the likelihood of a >1 step response in the during CFL. The horses shifting from IS to NIS performing significantly fewer >1 step responses than the horses shifting from NIS to IS (*F*_1,356_ = 18.68, *p* < 0.000001. There were no effects of treatment, task-type change or task-type*treatment interactions on the duration of the CFL session (additional data and statistics in Supplementary Materials).

HRs were not significantly different for the change in task-type (IS to NIS task: 48.22 [37.58–58.87 bpm 95% CI], NIS to IS task: 49.60 [38.78–60.42 bpm 95% CI] *F*_1,8_ = 0.067, *p* = 0.80) and treatment groups (C: 45.64 [36.20–55.08 bpm 95% CI], S: 52.19 [41.50–62.87 bpm 95% CI] *F*_1,8_ = 1.15, *p* = 0.25) and there was no significant task-type*treatment interaction (*F*_1,8_ = 2.77, *p* = 0.13). There was no significant difference in the cortisol concentrations resulting from the change in task-type (IS to NIS task: 0.77 [0.66–0.89 ng/mL 95% CI], NIS to IS task: 0.67 [0.43–0.90 ng/mL 95% CI], *F*_1,8_ = 0.90, *p* = 0.371) or the treatment (C: 0.73 [0.57–0.89 ng/mL 95% CI], S: 0.71 [0.49–0.93 ng/mL 95% CI], *F*_1,8_ = 0.27, *p* = 0.874), or a significant treatment*task-type interaction (*F*_1,8_ = 1.89, *p* = 0.21). There were also no significant differences in BDNF levels based on treatment group (C: 10.80 [8.06–13.54 pg/mL 95% CI], S: 12.64 [3.60–21.68 pg/mL 95% CI], *F*_1,6_ = 0.21 *p* = 0.665), direction (IS to NIS task: 11.26 [9.68–12.84 pg/mL 95% CI], NIS to IS task: 12.18 [2.74–21.63 95% CI], *F*_1,16_ = 0.02, *p* = 0.883), or treatment group* task-type interaction (*F*_1,16_ = 0.71, *p* = 0.433).

### 3.5. Comparison of CFL to OL1

The OL1 session and the CFL phase both involved novel learning tasks (original acquisition of the response-OL1 and the change in task-type during CFL) and consequently, they were compared to evaluate if there were differences in the learning outcomes as well as physiological responses. Both task groups achieved the same basic level of accuracy during the CFL as they did in the OL1 demonstrating it was experienced as novel learning task despite the similarities in the biomechanics of the response, confirming the lack of stress effects on CFL acquisition (*F*_1,596_ = 1.41, *p* = 0.24, supplementary Figure S5). There was evidence that the OL partially facilitated acquisition of the CFL learning as there was a significant increase in >1 step locomotory responses (*F*_1,596_ = 25.71, *p* = 0.000005 supplementary Figure S6), and a significant decrease in the number of taps applied in the CLF compared to the OL1 (*F*_1,596_ = 9.19, *p* = 0.002, supplementary Figure S7) meaning all horses were quicker to respond to the whip taps. The number of taps applied did not significantly differ between the task-types despite the direction change. However, while the number of taps applied to horses that initially learnt the IS task during OL did not differ between OL1 and the CFL, the horses that initially learnt the NIS task in OL, had half the number of taps applied during the reversal from the NIS task to the IS task in the CFL (direction* phase interaction *F*_1,656_ = 19.27, *p* = 0.000008. There were no significant differences in HR between OL1 and CFL, however salivary cortisol was significantly higher during OL1 than the CFL (*F*_1,20_ = 19.98, *p* = 0.001) and the BDNF concentrations of the NIS horses was significantly higher during OL1 (*F*_1,16_ = 4.92, *p* = 0.041, supplementary Figure S8). There were no other significant effects based on the task-type or phase (additional data, statistics and supplementary Table S4 in Supplementary Materials).

### 3.6. Relationships between Learning Outcomes and Physiological Indicators (Spearman’s Correlations)

During the OL, there were no significant associations between correct responses and physiological measures, however HR and BDNF were moderately associated (*r_s_* 0.584, *p* = 0.007). During treatment, BDNF was moderately associated with cortisol (*r_s_* 0.477, *p* = 0.008) as was HR (*r_s_* 0.395, *p* = 0.017). During CLF, cortisol was negatively associated with correct responses (*r_s_* −0.604, *p* = 0.038) and HR was associated with cortisol concentrations (*r_s_* 0.621, *p* = 0.031). None of the other relationships were significant, (correlation matrix tables in supplementary Tables S5–S8).

## 4. Discussion

This study provides experimental evidence of what is observed anecdotally, that training tasks that do not align with the horse’s ethology are learned more slowly and require more trials to achieve habit-like characteristics. Contrary to expectations, the repeated stress exposure which induced higher HRs and increased salivary cortisol concentrations, did not affect the acquisition of the cognitive flexibility task. However, the stress exposed horses were sensitised to the aversive characteristics of the training stimulus during the CFL but this did not translate into more correct responses during the CFL. Irrespective of treatment group, higher salivary cortisol concentrations were associated with reduced learning acquisition in the CFL task, a finding that mirrors a previous study [84]. Neither the relative difficulty of the OL or CFL tasks or the stress treatment significantly affected serum BDNF concentrations, however we do report primary evidence of an association between serum BDNF and HR and salivary cortisol in horses. The possible neural correlates for the results are discussed.

### 4.1. Differences in OL and IS-NIS Task-Type Acquisition

Although the two learning tasks in this study were biomechanically and energetically identical, the speed at which they were acquired varied significantly depending on the alignment of the task-type with equine ethology. The slower acquisition of the NIS task and the IS-NIS shift in the CFL may be due to the inherent conflict between the task and the ethology of horses, that is, they were required to approach an aversive stimulus that they would normally avoid. In natural contexts, escaping or avoiding potentially harmful stimuli confers fitness and a range of neural processes facilitate behavioural responses. When an aversive stimulus is perceived, a neural ensemble consisting of the periaqueductal grey, amygdala and lateral habenula controls innate behavioural responses to the stimulus precipitating defensive behaviour such as freezing, flight or aggression [85,86]. In addition, when activity in the defensive circuit is heightened, activity in the prefrontal cortex and dorsomedial striatal regions which flexible selection of actions is inhibited, potentially impairing novel learning [87,88]. It may be that the NIS horses experienced conflict between their SSDR (to move away from the US-a response that was punished) and the response they had to perform to actually escape or avoid it (move towards it) that interfered with quickly learning the correct response. In active avoidance learning studies in rodents, high levels of defensive behaviour such as freezing may completely prevent the animals learning the avoidance task [89] because the defensive behaviour (freezing), is incompatible with the active avoidance behaviour (locomotory). The greater likelihood of the OL NIS task-type and IS-NIS task switch horses performing smaller locomotory responses also suggests a reluctance to make an approach response to the US, despite receiving punishment for not doing so. In comparison, the OL IS task-type and NIS to IS task switch horse’s response aligned with their ethology so was acquired more efficiently and they reached higher levels of accuracy. Because the stimulus was applied to the right side of the horse only, differences in performance are not likely to be due to variability associated with brain laterality or differences in familiarity with handling on different sides as reported elsewhere [90,91,92].

The learning task induced an increase in HR for both groups, although not to levels likely to induce a large increase in noradrenaline sufficient to exert strong influences on learning [93,94]. The NIS horses’ HRs peaked in OL1 and the IS horses peaked in OL2 2, thereafter the HRs declined, whereas there was no significant difference in salivary cortisol or serum BDNF concentrations. The decline in HRs over the OL sessions provides evidence that as the horses gained control over the US by either escaping or even avoiding it, and there was a reduction in sympathetic nervous system activation [64].

Our results align with industry reports and provide experimental evidence to trainers of the necessity of adapting techniques when training horses to perform tasks that have the potential to elicit instinctive escape responses can then lead to dangerous and hard to control SSDRs such as rearing or bolting. Learning tasks that require horses to approach or habituate to aversive stimuli or places have the potential to activate the defensive neural network which may simultaneously impair activity in brain regions necessary for learning the response including the fronto-cortical-dorsomedial striatal loop [95]. Training should therefore be adapted to minimise fear and escape responses during training by minimising the horse’s exposure to aversive training stimuli, allowing for additional time to complete the training or including techniques such as positive reinforcement which can enhance dopamine release and associated synaptic plasticity and consequently learning [96]. In addition, our results demonstrate that even where horses have developed habit-like responses to approaching aversive stimuli, they can rapidly learn to avoid the same stimuli in the right circumstances, likely due to the influences of the defensive neural network and associated neurotransmitter release that facilitates escape behaviours [97]. This finding provides experimental evidence for the development of unwanted avoidance and escape behaviour in horses, for example that have previously been trained to load onto transport vehicles. By changing the contingency, we were able to rapidly extinct the approach response and replace it with its opposite. Trainers and owners should be aware that even when a well-trained, non-instinctual response appears to be habitual, if the trainer is not consistent with cues and the timing of their release, they may inadvertently introduce unpredictability into the training context leading the horse to trial new behaviour that may include escape/avoidance rather than approach of the inherently aversive stimulus or place. If these responses are then repeated they can develop into a problem behaviour that may require retraining [18].

### 4.2. Lack of Stress Effect on Cognitive Flexibility Learning

There are a number of possible reasons for the lack of stress effects on the CFL task. During aversive learning, the activity of prediction coding dopamine neurons [98] is attenuated when an unexpected aversive outcome is registered generating a teaching signal to motivate the organism to adapt to the changed contingency [99,100,101]. In the CFL, responses that had previously enabled the horses to either escape or avoid the aversive training stimulus were punished representing an unexpected aversive consequence. Dopamine efflux during aversive learning is believed to register both the valence (positive or negative) [100] and value (low to high) [102] of avoiding aversive stimuli. During the CFL session, the likely decrease in dopamine release from neurons that code aversive prediction errors and the high value of rapidly learning to escape the whip stimulus would have generated a strong teaching signal [37] to facilitate the acquisition of the new response. Wendler et al. [103] successfully extinguished habitual avoidance responses in rats by presenting conditioned and unconditioned aversive stimuli non-contingently to the avoidance responses of the subjects. Chronic stress has been implicated in attenuated dopamine signalling from reward sensitive ventral tegmental neurons and impaired learning, [54], however our data does not show evidence of this effect. Consequently it is plausible that aversive dopamine prediction errors contributed to the acquisition of the task irrespective of the extrinsic stress treatment.

A second possible explanation relates to the potential role of metaplasticity or memory priming [104]. Metaplasticity or priming processes within learning related dendritic spines associated with prior learning can facilitate the acquisition of new learning, although the precise mechanisms are still to be elucidated (reviewed in [105]). It is plausible that OL related synaptic plasticity in the relevant neural ensembles facilitated the acquisition of the CFL, task, specifically, performing a locomotory response to the whip stimulus. There were significantly fewer taps applied and significantly more >1 step responses in the CFL than the OL1 even though the number of correct responses was not significantly different. This finding departs from other equine studies which reported no effect of prior learning on performance in different (e.g., Fenner et al. [91]) or similar (Ahrendt et al. [90]) aversive instrumental learning tasks within the same training session. Compared to our study, the lack of learning facilitation reported in Arendt et al. [90] and Fenner et al. [91] could be due to the timing of the second tasks in these studies, which took place during the very early consolidation processes of the first task when new learning related synaptic plasticity activity is still labile and vulnerable to disruption [106]. The CFL session in this present study occurred after the memories of the original task were likely consolidated to a stable form [107] and consequently, possibly via the contribution of metaplasticity processes, the acquisition of the CFL was facilitated irrespective of the stress treatment.

A third factor may be the timing of the CFL learning task relative to the stress exposure. There was a 24 h gap between the final stress exposure and the CFL session, meaning that any potential cortisol mediated impairment of acute, non-genomic cortisol activity on learning had likely dissipated prior to the learning of the CFL task [108]. There was no lasting effect of the stressor on peripheral cortisol concentrations of the stress exposed horses, suggesting the stressor duration and intensity was not sufficient to elicit the widespread organisational or functional changes to learning regions such as have been reported in rodent studies and which can impair cognitive flexibility in appetitive instrumental and spatial learning tasks [3,109,110,111]. The repeated stress treatment implemented in this study, while causing sympathetic nervous system and HPA axis activation, neither impaired nor facilitated cognitive flexibility acquisition during the CFL.

### 4.3. Proxies for Neurotransmitter Release and Activity

There were no correlations between physiological indicators and the acquisition of the OL tasks, however we report a moderate negative association between cortisol concentrations and the performance of correct responses during the CFL. This mirrors a previous finding in horses where higher salivary cortisol concentrations were associated with slower acquisition of a similar negatively reinforced locomotory response [84]. However, as the cortisol concentrations immediately after the CFL were lower than those of the OL phases, the negative association between cortisol concentration and learning performance appears to reflect intrinsic cortisol reactivity in the horses rather than a task-induced effect. Cortisol phenotypes have been detected in other species [112] and in humans and rodents, correlations between impaired learning and glucocorticoid concentrations have been identified [113]. Smeets et al. [114] reported impaired cognitive flexibility in humans with higher salivary cortisol concentrations irrespective of stress exposure. To date, while breed differences in salivary cortisol have been identified [115], there is little data on whether cortisol genotypes per se exist in horses. Further research evaluating cortisol reactivity and cognition in horses is required to confirm our finding as other studies have reported either no relationship between salivary cortisol and task performance [116] or a transient increase in performance in an appetitive task acquisition in horses [117].

The increased salivary cortisol concentrations during the stress treatment were positively correlated with both HR and BDNF, however there was no treatment effect on BDNF concentrations when assessed in isolation. Higher levels of BDNF in the amygdala are associated with higher levels of cortisol in this region in other species [118], and it appears that horses experiencing a stronger activation of the HPA axis as indicated by cortisol concentrations, also had higher BDNF concentrations, a novel finding in horses. BDNF release and activity in response to stress exposure is heterogeneous and its specific roles in individual brain regions involved in learning as well as adaptation to stress continues to be elucidated [119,120]. Little is known about BDNF in horses. Our pilot testing revealed that serum BDNF concentrations in horses is substantially lower than for humans or rats. Kongoun et al. [69] analysed serum BDNF and reported that a 12 month exercise program increased resting serum BDNF levels compared to sedentary horses. BDNF is released from neurons in an activity dependent manner and the timing of peak release differs depending on whether the activity involves stress responses or exercise, with responses to stress leading to later peaks than exercise [121,122]. The stress exposed horses in this study experienced considerable psychosocial stress during the treatment but were only were physically during the NO and MS, but not SI. It is not clear that the BDNF samples were collected at the most opportune time to capture changes in BDNF in response to the stress treatment. Further research could compare BDNF levels in equine serum across different collection periods.

There was no lasting effect of the stress induced increases in HR (and by proxy noradrenaline) and salivary cortisol on acquisition of the CFL task, which differs from findings in chromic stress protocols in rodents [123]. Daily stress exposure of more than 10 days duration that leads to elevated concentrations of cortisol in the brain is associated with impaired cognitive flexibility learning and structural changes [109,124] in brain regions such as the basolateral amygdala which is essential for extinction learning [125]. Six days of daily stress exposure was chosen for this study to as reflect of a typical working week for horses in training. The stressors were chosen to mimic the kinds of stressors that horses may encounter in their daily lives, however our results suggest that this duration is insufficient to impair cognitive flexibility acquisition. Future studies, utilising convenience samples of horses already exposed to industry-based chronic or repeated stress could further explore this issue.

### 4.4. Limitations

The findings of this preliminary study require replication. For instance horses sent for retraining would likely have performed considerably more repetitions of the unwanted habit prior to commencing retraining than the 100 repetitions undertaken during the OL. It is also likely that any retraining procedure would involve considerably more repetitions of the new response. Further studies could explore whether these results are replicated in horses exposed to a greater number of original learning type trials that mimic industry conditions more closely.

### 4.5. Conclusions

While preliminary, this study presents evidence that even where the learning tasks share identical biomechanical and energetic characteristics, those that align with the horse’s natural escape behaviour are learned more efficiently and become habit-like more quickly than tasks which do not. These results also suggest that when retraining responses that require cognitive flexibility, trainers should take into account the fact that for the horse the learning is likely to be perceived as novel and adjust their expectations about how the horse experiences the procedure. This is particularly important in the early sessions where habit responses undergo positive punishment, a form of uncontrollable and unpredictable stress, until the horse acquires the new response that enables it to escape the aversive stimuli. Trainers should be aware that tasks that elicit activity in the defensive neural networks may be harder to learn due to inhibition of activity in the flexible goal-directed networks as well as increased in activity in the habit network that may impair extinction of unwanted habits. This has particular relevance when the training task involves retraining habitual avoidance of stimuli perceived to be aversive such as shying at frightening objects or unwillingness to enter transport vehicles.

## Figures and Tables

**Table 1:** Horse details.

Horse	Sex	Age	Breed	Original Task-Type Group	Treatment Group
1	Mare	7	Thoroughbred	Instinctual	Stress
2	Mare	7	Thoroughbred	Non-Instinctual	Control
3	Mare	14	Thoroughbred x Riding pony	Instinctual	Control
4	Mare	17	Quarter Horse	Non-Instinctual	Stress
5	Gelding	13	Welsh Pony	Instinctual	Stress
6	Gelding	16	Australian Stockhorse	Non-Instinctual	Control
7	Mare	9	Welsh pony	Instinctual	Control
8	Mare	13	Welsh Pony	Non-Instinctual	Stress
9	Mare	7	Thoroughbred	Instinctual	Control
10	Gelding	11	Pony	Non-Instinctual	Stress
11	Mare	14	Australian Stock Horse	Instinctual	Stress
12	Gelding	17	Paint/Quarter Horse	Non-Instinctual	Control

## Data Availability

The raw data is available on request.

## Supplementary Materials

The following supporting information can be downloaded at: https://www.mdpi.com/article/10.3390/ani12202818/s1: Table S1: Details of stress and control treatments; Table S2: Binary codes and definitions for Generalised Linear Mixed Models with binary logistic regression; Table S3: Pre-test physiology paired samples *t*-tests; Table S4: Comparison of HRs during the first original learning session and the cognitive flexibility learning session; Table S5: Original learning Spearman’s Correlations; Table S6: Treatment Spearman’s Correlation; Table S7: Cognitive flexibility learning Spearman’s Correlations; Figure S1: Predicted probability of horses making > 1 Step responses during the four original learning sessions (OL); Figure S2: Mean original learning session durations (s), OL = original learning. Letters differ significantly at *p* < 0.0001, between sessions; Figure S3: Mean taps/trial per original learning session (both task-types); Figure S4: Mean taps/trial during the response shift; Figure S5: Comparison of the mean predicted probability of a correct response during the first original learning session and the cognitive flexibility learning session; Figure S6: Mean predicted probability a > 1 step response occurring in the OL1 session compared to the response shift session; Figure S7: Mean taps/trial comparing the first original learning session to the cognitive flexibility session session; Figure S8: Mean BDNF concentrations (pg/mL) between the first original learning session and the cognitive flexibility session.
