# Supplementary material for: Habit Formation and the Effect of Repeated Stress Exposures on Cognitive Flexibility Learning in Horses
Source: Animals (Basel). 2022 Oct 18;12(20):2818. doi: 10.3390/ani12202818 (PMC9597801; doi:10.3390/ani12202818)
Supplement: Supplementary file 1 [file animals-12-02818-s001.zip › animals-1946287-supplementary.pdf]

Supplementary materials

Habit formation and the effect of repeated stress exposures on cognitive flexibility learning in horses

C. Henshall*, H. Randle, N. Francis, R. Freire

School of Agricultural, Environmental and Veterinary Sciences

Charles Sturt University, Wagga Wagga, NSW Australia

Corresponding author: *chenshall@csu.edu.au

Materials and methods supplementary materials

Table S1: Details of stress and control treatments

**Treatment Group**	**Days**	**Stressor**	**Description**
Control	1-6	Control	Pairs of control horses were tethered in visual contact with each other and were left alone for the 15 minutes of the treatment. They were observed by an experimenter from a distance of approximately 50 m. The control horses could see the observer.
Stress	1 and 3	Social Isolation	The horse was led to SI location on its own and released into the yard. The handler retreated to a location that enabled visual contact with the horse but was shielded from the view of the horse.
	2 and 4	Novel objects	The horse was released into the roundyard and the NOs were introduced and manipulated by an experimenter inside the yard. They were manipulated in response to the horse's behaviour (looking, moving away, moving towards them, speed of movement, postural indicators of stress) and moved towards, away or kept stationary. The balls were rolled, bounced and tapped. The timing and intensity of the ball manipulation was such that the horse was not reinforced for any specific behaviour and to reduce the likelihood of habituation. Fig. 1.1.
	Days 3 and 6	Multimodal stressors	The horse was released into the roundyard and the handler exited. The experimenters stood outside the roundyard to manipulate the RCC and amplifier. The MS exposure involved

			five minutes of RCC, three minutes of sounds, five minutes RCC and two minutes of the sounds. During the playing of the sounds the RCC was driven to the side of the roundyard and remained stationary. The movement of the RCC was manipulated in response to the horse's behaviour using the same criteria as the NOs Fig. 1.2, main text.

Statistical analysis supplementary materials

Table S2: Binary codes and definitions for Generalised Linear Mixed Models with binary logistic regression

**Phase(s) or session**	**Variable**	**Description**	**Code "0"**	**Code "1"**
**Original learning sessions and Cognitive flexibility learning**	Response correctness	Presence or absence of a correct response	Absence of correct response	Presence of correct response
	Size of locomotory response	Presence or absence of a locomotory response of any kind >1 step	Absence of a locomotory response >1 step	Presence of a locomotory response >1 step
**Original learning session 1 versus cognitive flexibility learning session**	Response correctness	Presence or absence of a correct response	Absence of correct response	Presence of correct response
	Size of locomotory response	Presence or absence of a locomotory response of any kind (correct/incorrect) >1 step	Absence of a locomotory response >1 step	Presence of a locomotory response >1 step

Results supplementary materials

Pre-test physiology

Table S3: Pre-test physiology paired samples *t-tests*

Measure	Group	Mean	95% Confidence Interval of the Difference		t	df	Sig. (2-tailed)
			Lower	Upper			
HR	IS vs NIS	3.61.	-3.15	10.36	1.37	5	0.228
	Control vs Stress	-2.37	-8.16	7.69	-0.07	5	0.942
Cortisol	IS vs NIS	.02167	-.26515	.30848	.194	5	0.854
	Control vs Stress	.10167	-.16144	.36477	.993	5	0.366
BDNF	IS vs NIS	1.99600	-6.98632	10.97832	.617	4	0.571
	Control vs Stress	-2.94000	-11.86774	5.98774	-.914	4	0.412

Original learning

Probability of a >1 step response during original learning sessions

There was a significantly higher probability of a >1 step response in the later OL sessions than the first two sessions ($F_{3,1190}$ =87.58, p<0.000001) and IS horses had a significantly higher probability of making >1 step responses than NIS horses across the four sessions ($F_{1,1190}$ =8.27, p=0.004), however by the final session, the probabilities were equal (session*task-type $F_{3,1190}$ =6.06, p=0.0004, Figure S1).

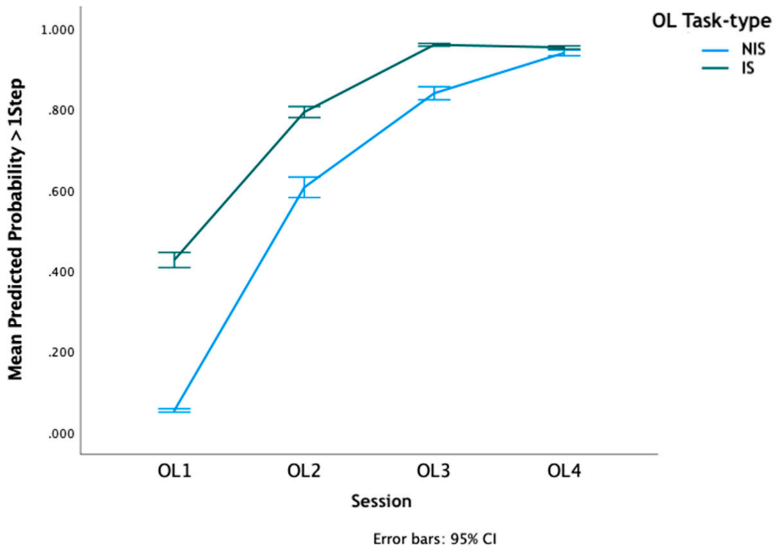

Figure S1: Predicted probability of horses making >1 Step responses during the four original learning sessions (OL).

Duration of original learning sessions

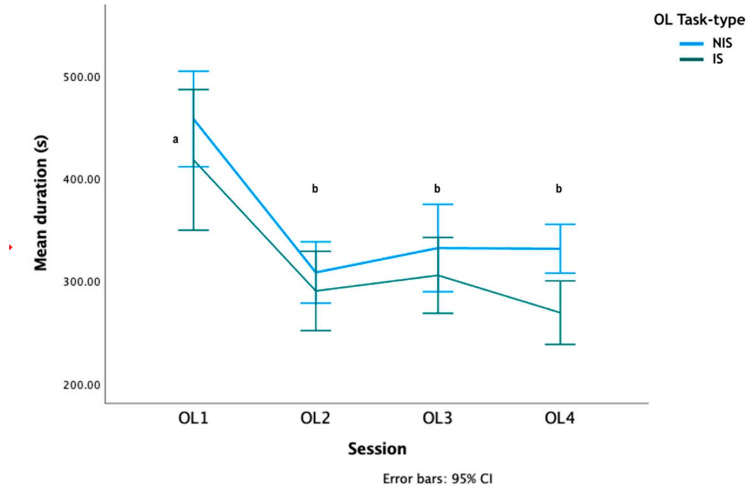

Figure S2: Mean original learning session durations (s), OL= original learning. Letters differ significantly at p<0.0001, between sessions.

Taps applied during original learning

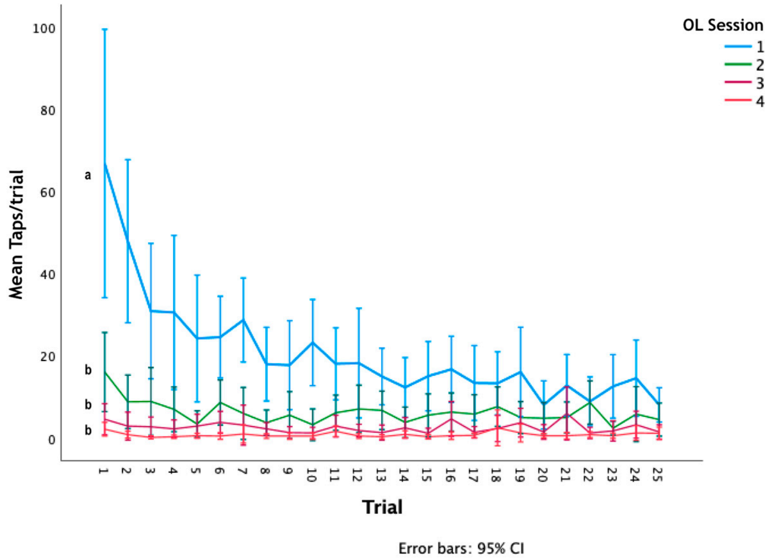

Figure S3: Mean taps/trial per original learning session (both task-types). Letters which differ, differ significantly at p<0.0001 between sessions.

Cognitive flexibility learning

Taps applied during the cognitive flexibility learning session

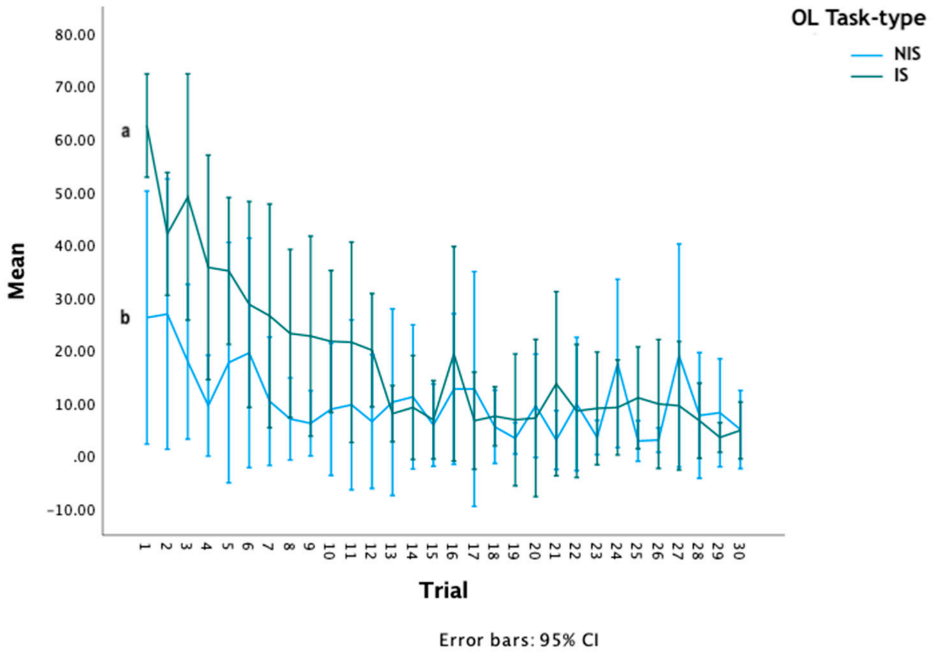

Figure S4: Mean taps/trial during the cognitive flexibility learning (CFL) session. OL=original learning task-type, NIS= horses who changed from NIS task to IS task during the CFL, IS=horses who changed from the IS task to NIS task during the CFL.

>1 Step responses during cognitive flexibility learning session

During the CFL, the horses changing from IS task to NIS task performed significantly fewer >1 step responses than the horses changing from NIS task to IS task (IS-NIS: 6.17 ±7.52, NIS-IS 19.93 ± 8.45, $F_{1,356}$ =18.68, p<0.000001). The treatment did not have a significant effect, (C: 13.17 ±11.97, S: 12.83 ± 9.83 ,$F_{1,356}$ 0.06, p=0.810) but there was a significant task-type*treatment interaction with C IS-NIS horses making fewer >1 step responses than the S IS-NIS horses whereas the S NI-IS horses made fewer >1 step responses than the C NIS-IS horses (C IS-NIS:4.67 ± 7.23, C NIS-IS:21.67 ± 9.45, S IS-NIS:7.67 ± 9.07, S NIS-IS:18.00 ± 8.89, $F_{1,356}$ =5.76, p=0.017).

Duration of cognitive flexibility learning session

The duration of the CLF sessions was similar for task-type (IS-NIS: 476.100 ± 121.53 s, NIS-IS: 419.60 ± 36.00 s, $F_{1,20}$ =2.21, p=0.153), and treatment (C: 458.90±236.77s, S:436±3835s, $F_{1,20}$ =0.34, p=0.568). The task-type*treatment was also non-significant ($F_{1,20}$ =0.01, p=0.903).

Comparison of cognitive flexibility learning session to first original learning session

Comparison of the probability of correct and >1 step responses between OL1 and CFL sessions

During the CFL the predicted probability of a correct response was slightly higher compared to the first original learning session (OL1) but not significantly different, ($F_{1,596}$ =1.41, p=0.236, Figure S5), demonstrating that the horses reached the same basic level of performance as occurred in OL1 (Mean count of correct responses for both task type groups: OL1: 4.5± 5.42 correct responses/session, CFL: 5.250± 4.69 correct responses/session, ($F_{1,596}$ =1.41, p=0.236). There was a significant difference in the probability of a correct responses based on task-type with the IS horses having a higher probability of performing more correct responses across the two sessions than the NIS horses ($F_{1,596}$ =7.14, p=0.008). There was a significant phase* task-type interaction ($F_{,1,596}$ =51.53, p<0.000001), with IS horses having a higher probability of performing correct responses in OL1 than NIS horses, whereas the situation was reversed in the CFL, with horses changing from NIS to IS having a higher probability of performing more correct responses than horses changing from IS to NIS (Figure S5, letters indicate significant differences).

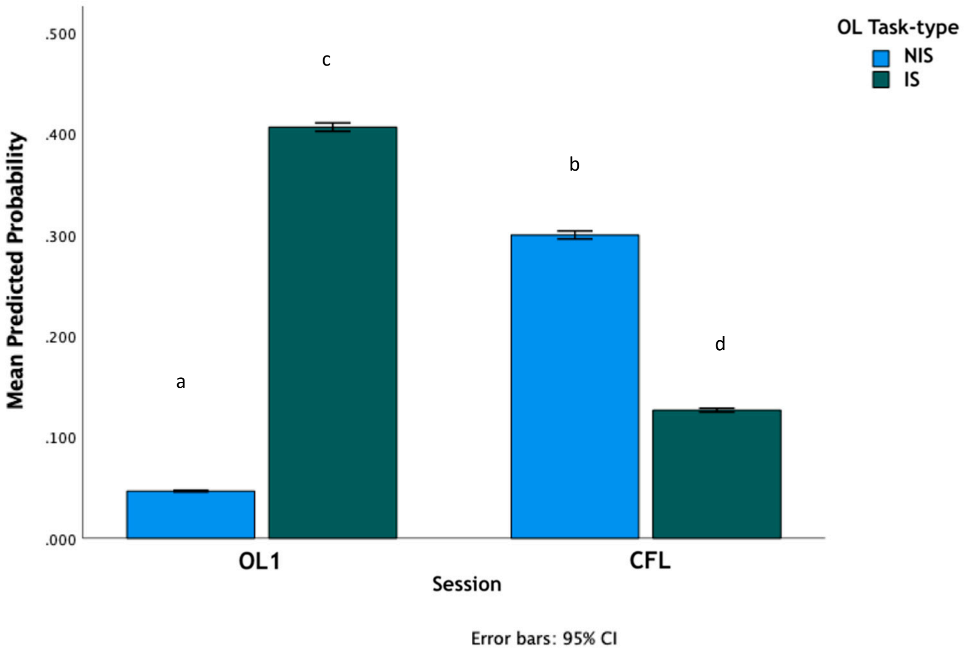

Figure S5: Comparison of the mean predicted probability of a correct response during the first original learning session (OL1) and the cognitive flexibility learning session (CFL). The task-type label (IS or NIS) refers to the OL task-type. During the CFL, the response task-type was changed so responses in the CFL were in the opposite task-type to the OL task-type. Y-axis-mean predicted probability of correct response. Letters: a + b differ significantly between OL1 and CFL, c + d differ significantly between OL1 and CLF at p=<0.001).

There was a higher probability of >1 step responses during the CFL compared to OL1 ($F_{1,596}$ =25.71, p=0.000005, Figure S6), and the NIS-IS horses had a significantly higher probability of performing a >1 step responses in the CFL than the IS horses during OL1 and the CFL ($F_{1,596}$ =96.29, p<0.000001). The task-type did not significantly differ ($F_{1,596}$ =0.48, p=0.491).

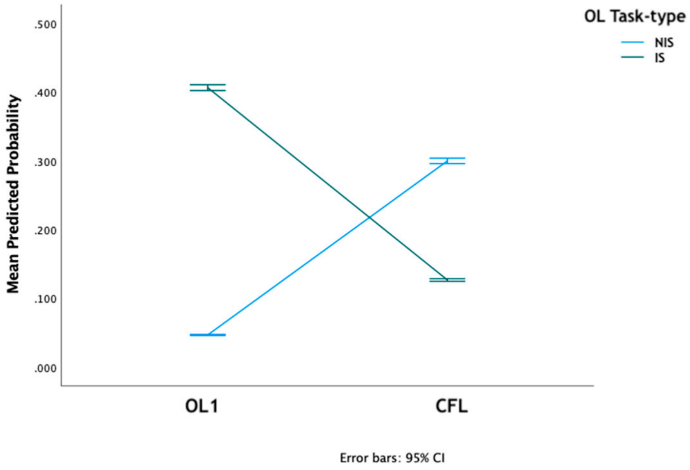

Figure S6: Mean predicted probability a > 1 step response occurring in the OL1 session compared to the cognitive flexibility learning session. OL=original learning, CFL=cognitive flexibility learning, IS= instinctual task-type during OL, NIS =non-instinctual task-type during OL.

Comparison of taps applied and duration of sessions between OL1 and CFL sessions

There were significantly fewer taps per trial applied during the CFL, than in the OL1, (OL1: 20.65 [18.06-23.24 95%CI], CFL: 15.69 {13.69-17.70 95%CI], $F_{1,596}$ =9.19, p=0.002, supplementary fig. 7), but no significant differences based on task-type (IS: 18.43 [14.91-21.95 95%CI] NIS: 22.76 [19.78-25.72 95%CI], $F_{1,596}$ =1.73, p=0.189, Figure S7) meaning the number of taps applied during OL1 and the number of taps applied during the CFL did not differ in spite of the task-type change. However, the task-type of the change significantly influenced the number of taps applied in the CFL for the OL-NIS horses, who then changed to the IS task-type in the CFL. They had less than half the number of taps applied in the CFL compared to OL1, whereas the number of taps applied to the IS horses who changed to NIS during the CFL, did not differ between OL1 and the CFL task-type* phase interaction ($F_{1,656}$ =19.27, p=0.000008).

The OL1 session was a similar duration to the duration of the first 25 trials in the CFL ($F_{1,20}$ =0.13, p=0.724), and there was no difference based on task-type ($F_{1,20}$ =0.056, p=0.813) or phase*task-type ($F_{1,2}$ =2.996, p=0.099).

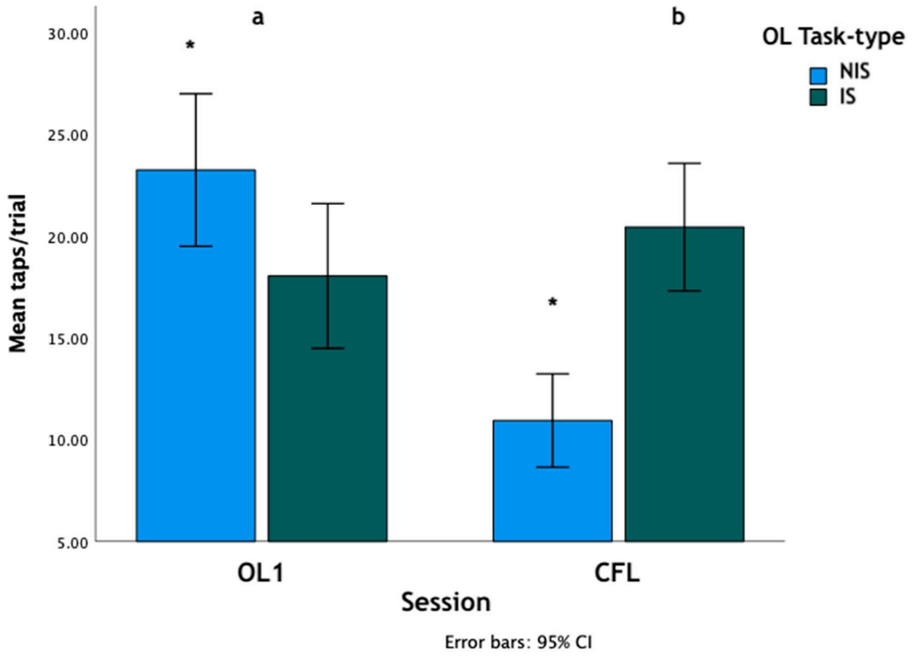

*Figure S7: Mean taps/trial comparing the first original learning session to the cognitive flexibility learning session. OL1=original learning 1, CFL= cognitive flexibility learning, IS= instinctual task-type during OL , NIS=non-instinctual task-type during OL. Letters which differ are significant at p<0.01 between phases.**

*different at p<0.001 between phase*task-type. Bars without letters are not significantly different between treatment or task-type groups.*

Comparison of physiological responses between OL1 and CFL sessions

There was no significant difference in HRs, between OL1 and the CFL, ($F_{2,19}$ =0.960, p=0.18) and nor were there were significant differences between the learning directions ($F_{1,18,}$ 1.72, p=0.207) or a significant task-type*phase interaction ($F_{1,18}$ =2.14, p=0.16,Table S4).

Table S4: Comparison of HRs during the first original learning session and the cognitive flexibility learning session.

Phase	OL Direction	Mean HR (bpm)	95.0% Lower CL	95.0% Upper CL
OL1	NIS	48.47	41.07	55.86
	IS	40.65	38.30	42.99
CFL	NIS	49.97	35.70	64.23
	IS	48.22	37.58	58.87

OL=original learning session 1, CFL= cognitive flexibility learning session.

During OL1 cortisol was higher than during the CFL (Mean ng/ml-OL1: 0.99 [0.93-1.04 95% CI] CFL: 0.72 [0.61-0.83 95% CI] $F_{1,20}$ =23.54, p=0.0001) but there was no difference based on task-type, ($F_{1,20}$ =0.07,p=0.802) or a significant phase*task-type interaction ($F_{1,20}$ =2.66, p=0.12). The NIS horses had higher BDNF levels than the IS horses during OL1 compared to the CFL session ($F_{1,16}$ =4,92, p=0.04, Figure S8), however BDNF concentrations did not significantly differ between the two phases or the task types, (phase: $F_{1,16}$ =1.87, p=0.19, task-type: ($F_{1,16}$ =0.35, p=0.19).

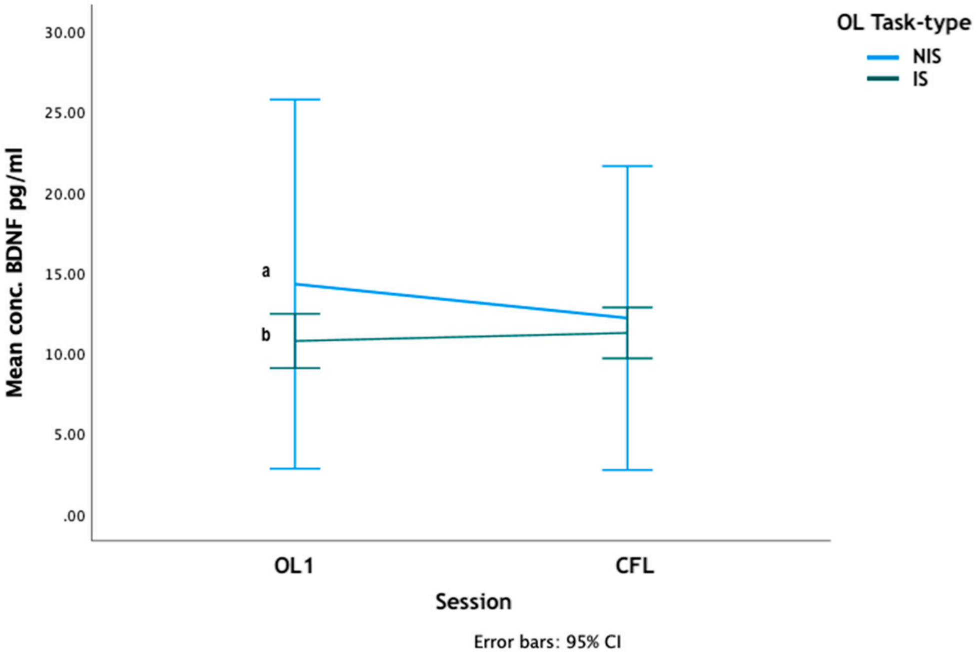

Figure S8: Mean BDNF concentrations (pg/ml) between the first original learning session and the cognitive flexibility learning session. OL=original learning, CFL= cognitive flexibility learning. IS= instinctual task-type during OL, NIS= non-instinctual task-type during OL. Letters which differ, differ significantly at p<0.05 between directions.

Spearman's Correlations

Table S5: Original learning

		BDNF	Cortisol	HR	Correct responses
BDNF	Correlation Coefficient	1	-0.247	.584**	-0.214
	Sig. (2-tailed)	.	0.294	0.007	0.357
Cortisol	Correlation Coefficient		1	-0.092	-0.277
	Sig. (2-tailed)		.	0.668	0.180
HR	Correlation Coefficient			1	-0.119
	Sig. (2-tailed)			.	0.141
Correct responses	Correlation Coefficient				1
	Sig. (2-tailed)				.

*Correct responses for sessions 1 and 4 to align with collection of BDNF and salivary cortisol. OL=original learning.** Significant at p=<0.01 (two tailed)*

Table S6: Treatment

		BNDF	Cortisol	HR
BNDF	Correlation Coefficient	1	.477**	0.098
	Sig. (2-tailed)	.	0.008	0.607
Cortisol	Correlation Coefficient		1	.395*
	Sig. (2-tailed)		.	0.017
HR	Correlation Coefficient		.	1
	Sig. (2-tailed)			.

*** Significant at p=<0.01, * significant at p=<0.05, (two tailed)*

Table S7: Cognitive flexibility learning

		BDNF	Cortisol	HR	Correct responses
BDNF	Correlation Coefficient	1	0.212	0.297	-0.207
	Sig. (2-tailed)	.	0.556	0.405	0.567
Cortisol	Correlation Coefficient		1	.608*	-.582*
	Sig. (2-tailed)		.	0.036	0.047
HR	Correlation Coefficient			1	-0.214
	Sig. (2-tailed)			.	0.504
Correct responses	Correlation Coefficient				1
	Sig. (2-tailed)				

. significant at p=<0.05 (two tailed) .*
